# Ethnopharmacological Assessment of Medicinal Plants Used against Livestock Infections by the People Living around Indus River

**DOI:** 10.1155/2014/616858

**Published:** 2014-12-03

**Authors:** Sakina Mussarat, Rahila Amber, Akash Tariq, Muhammad Adnan, Naser M. AbdElsalam, Riaz Ullah, Roqaia Bibi

**Affiliations:** ^1^Department of Botany, Kohat University of Science and Technology, Kohat 26000, Pakistan; ^2^Department of Zoology, Kohat University of Science and Technology, Kohat 26000, Pakistan; ^3^Riyadh Community College, King Saud University, Riyadh 11437, Saudi Arabia; ^4^Department of Chemistry, Government College Ara Khel, Frontier Region Kohat 26000, Pakistan

## Abstract

The present study was aimed to document detailed ethnopharmacological knowledge of medicinal plants against livestock infections of an unexplored remote region of Pakistan. Semistructured questionnaires were used for data collection. Total 43 plants belonging to 26 families were found to be used in ethnoveterinary practices. Seeds (29%) were found to be the most frequent plant part used followed by leaves (22%). Ethnoveterinary recipes were mostly prepared in the form of decoction and powdering. Informant consensus factor (Fic) results revealed high consensus for gastrointestinal (0.81), mastitis (0.82), and dermatological infections (0.80). *Curcuma longa* ranked first with highest fidelity level (FL) value (66%) followed by *Trachyspermum ammi* that ranked second (58%). Preference ranking (PR) results showed that *Zingiber officinale*, *Punica granatum*, *Triticum aestivum*, *Gossypium hirsutum*, and *Withania coagulans* were the most preferred species for the treatment of diarrhea. Direct matrix ranking (DMR) results showed that *Morus alba*, *Melia azedarach*, *Withania coagulans*, *Cassia fistula*, *Azadirachta indica*, and *Tamarix aphylla* were the multipurpose species of the region. We invite the attention of pharmacologists and chemists for further exploration of plants having high Fic, FL, and PR values in the present study. Conservation strategies should be adopted for the protection of multipurpose plant species.

## 1. Introduction

Medicinal plants are being used for the treatment of various livestock ailments by the local peoples since earliest times. It is a recognized fact that plants are an important source of ethnoveterinary medicines [1]. From the last decade, ethnoveterinary practices have gained tremendous importance due to the discovery of some effective ethnoveterinary products. Ethnoveterinary practices are more common in developing countries including Pakistan due to different socioeconomic factors [2].

Agriculture is Pakistan's largest and important sector of the economy, which contributes to 23.3 percent of the total gross domestic product (GDP) [3]. In agriculture sector livestock is very important and its population in Pakistan has increased up to 167.5 million heads, which is contributing 51.1% in an agriculture economy [4]. Due to high dependency on agriculture and livestock, Pakistan is the world's 5th largest milk producing country [5]. Approximately 53 million people of Pakistan reside in rural areas and mostly derive their income from livestock through different methods [6]. They have limited resources available for feeding to their livestock and use whatever is available, which ultimately leads to poor health livestock production resulting in economic losses. At present annual growth rate of meat and milk production in Pakistan is very slow due to poor forage quality, high incidence of diseases, poor prophylaxis, and high cost of modern veterinary drugs [7]. Majority of the Pakistani farmers own 4-5 numbers of livestock and it is very difficult for them to treat their animals with modern drugs due to high cost. Moreover issues like development of drugs resistivity in livestock and consumers unfriendly effects like high antibiotic residues in milk and other animal by-products increase the importance of ethnoveterinary medicines in addition to their significance in animal health care system [8]. Under such conditions traditional veterinary medicines provide a cheap therapy and easy accessibility as compared to modern veterinary drugs. It will also help in poverty alleviation by empowering peoples to use their own resources for treating livestock ailments [5]. That is why majority of the rural population in Pakistan is dependent on medicinal plants for the treatment of their animals based on their traditional knowledge. Despite the fact that traditional knowledge is very much important for the livestock health and productivity, the documentation of this knowledge is very much neglected in majority of the remote areas of Pakistan [9].

Present study was designed with the aim to document indigenous knowledge on ethnoveterinary practices of an unexplored remote region situated near Indus river of Pakistan. The area is surrounded by variety of remote regions of Pakistan such as Tank, South and North Waziristan, Bannu, and Lakki Marwat. Different ethnobotanical studies have been carried out in these adjacent areas even in the studied area due to higher dependency of tribal people on medicinal plants [10–15]. Despite having strong agricultural background of Pakistan very less attention has been given to these potential areas from ethnoveterinary point of view. The present study is the first attempt to explore detailed ethnoveterinary practices of this region of Pakistan where people have sound traditional knowledge and are highly engaged in utilizing ethnoveterinary practices for improving the health of their livestock and to compensate their income. Main objectives of the study are (i) to identify ethnoveterinary plants and detailed indigenous knowledge on herbal preparations; (ii) to identify plants with high bioactivity against specific ailments on the basis of informant consensus, fidelity level, and preference ranking; (iii) to identify candidate medicinal plants for further phytochemical and pharmacological investigation; and (iv) to identify multipurpose ethnoveterinary plants and factors responsible for their extinction in future using direct matrix ranking. The present study would be a great contribution in conserving valuable traditional knowledge on ethnoveterinary practices and provide baseline information for future* in vitro* and* in vivo* studies that could lead toward identification of novel active compounds and manufacturing veterinary drugs with low cost and fewer side effects.

## 2. Material and Methods

### 2.1. Study Area

**Figure 1:**
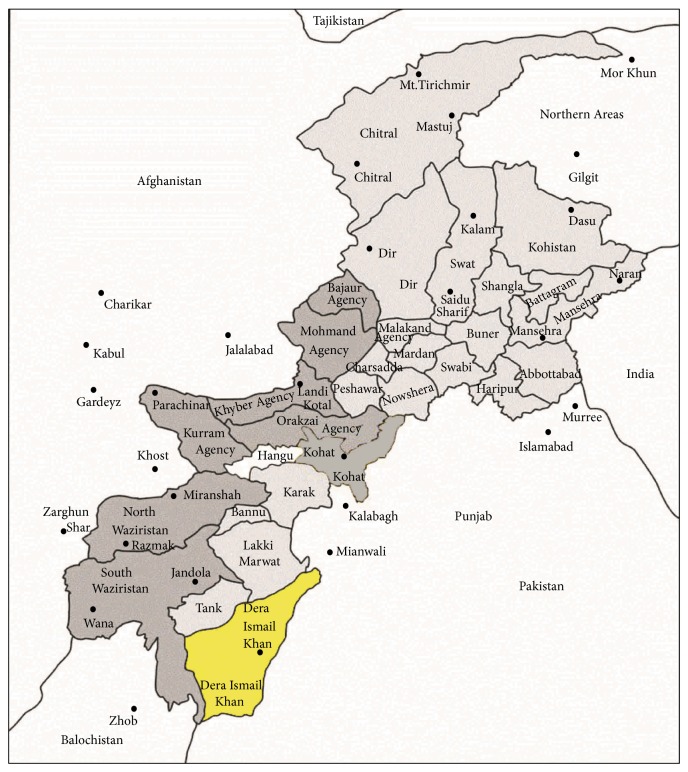
Map of the study area.

The present study was carried out in the Dera Ismail Khan often abbreviated as D. I. Khan, a district of Khyber Pakhtunkhwa province, Pakistan (Figure 1). D. I. Khan covers an area of about 7326 km^2^ and is situated between 70°.11′ and 71°.20′ E longitude and between 31°.15′ and 32°.32′ N latitude [16]. D. I. Khan has mostly flat dry plains, commonly called Daman, about 80 percent of the total area [11]. Aquatic and xerophytic vegetations are commonly grown in the study area [17].* Acacia modesta*,* Calotropis procera*,* Acacia nilotica*,* Eucalyptus camaldulensis, and Morus alba* are the dominant vegetation of the study area. The maximum and minimum temperatures recorded during summer and winter are 27°C to 42°C and 20°C to 40°C, respectively [11]. Mostly rainfall occurs in the late winter and early spring while in monsoon in June and July. Wheat, rice, sugar cane, dates, and variety of mangoes are produced in D. I. Khan. Among these, “Village Dhakki date” is the most famous product, not only used in the country but also exported to the Middle East, Europe, and United States. The area is rural in nature and inhabitants are very much dependent on livestock for economic and food purposes. Locals of the region use variety of medicinal plants for the treatment of livestock ailments due to expensive veterinary drugs.

### 2.2. Data Collection

Field work was carried out from May to August 2014. A total four field trips (each trip of 20 days) were made in each month (May, June, July, and August). Prior to data collection local representatives of the regions were visited and informed about the main theme of the study and to get their verbal consent for data collection and publication. The methods for the collection of data and voucher specimens during the field study followed that described by Martin [18]. Total 115 informants were selected on the basis of their traditional knowledge regarding livestock treatment in different villages of D. I. Khan. Ethical consent was taken individually from all the respondents by ensuring them that their traditional knowledge would be protected. This was done in order to acknowledge informants' cooperation in preserving the traditional knowledge of the study area and builds their confidence for providing reliable information. Out of 115 informants 90 were males and 25 were females. Age of the informants were ranged between 30 and 70 years old. Initially a questionnaire was designed and pretested with ten informants to identify the appropriateness for the data collection and later on modified according to the informants response. The modified questionnaire was then used to gather ethnoveterinary medicinal plants data of the study region from each informant individually. Informants were allowed to talk freely without any hesitation. Our final purpose was to get the complete list of medicinal plants used and/or known by each informant. All interviews were carried out in local language (*Saraiki*) of the study area. In addition, informants were divided into two groups and total three focus group discussions were also designed to gain further information on medicinal plants at the community level and to prove the reliability of data collected through semistructured interviews. Questionnaires designed to the respondents (traditional healers) about medicinal plants knowledge were mainly focused on local name of medicinal plant, types of disease treated, animal treated, remedy preparation, plant part used, use of single or mixture of plants for remedy preparation, mode of administration, dose requirement, recovery time, and usable duration regarding each medicine. The questionnaire also contained questions regarding general information of respondents such as name of the respondent, gender, age, education, and occupation.

### 2.3. Data Quality Assurance

During data collection each respondent was visited or contacted at least three times for the validity of information provided by them. In case of any deviation of respondent idea from the original information provided, it was rejected and considered irrelevant information. Only relevant information was subjected to further analysis process. Further data quality was ensured through proper training of data collectors, pointing out missing information, duplication of material, and careful analysis.

### 2.4. Data Organization and Analysis

Data collected from informants was organized using Microsoft Excel 2007 and Microsoft Word 2007. Informants were categorized into four age groups (30–40, 41–50, 51–60, and 61–70). Educational status of the informants was divided into five major classes (illiterate, primary, middle, secondary, and university). Occupation of both genders was categorized into five major fields, that is, housewives, shopkeepers, farmers, labor, and primary teachers. Plant habit was categorized into three classes, that is, herb, shrub, and tree. Plant parts were classified into leaves, stem, root, whole plant, seeds, buds, bulb, and fruit. Medicinal plants uses were categorized into 7 major categories, that is, gastrointestinal, dermatological, eye diseases, respiratory, reproductive, mastitis, and muscular. Recipes were classified into different groups, that is, decoction, powder, crushed, juice, paste, poultice, and infusion. Route of administration was divided into 3 categories, that is, oral, dermal, and nasal.

Informant consensus and fidelity level were used to verify the importance of medicinal plants.

### 2.5. Informant Consensus (Fic)

Informant consensus factor (Fic) was calculated on the reported cures of certain group of ailments. Within a community Fic designates the extensively used plants and helps in the selection of important medicinal plants for further pharmacological and phytochemical studies [19]. Reported veterinary problems were grouped into 7 major ailments. Fic values are high when a large number of respondents use one or few plants to treat a specific ailment, while Fic values are low when there is contradiction between informants regarding uses of plants [20, 21].

The Fic can be calculated by using the formula as follows:

(1) $$\begin{array}{c} \text{Fic}=\text{nur}-\frac{\text{nt}}{\text{nur}}-1, \end{array}$$

where Fic is the informants consensus factor, nur is the number of use citation in each category, and nt is the number of species used.

### 2.6. Fidelity Level (FL)

Fidelity level (FL) is useful for recognizing the most favored plants used for curing distinct livestock ailments by the respondents. FL values of highly preferred plants are greater than values of less preferred plants. FL values are always calculated in terms of informant's percentage claiming the use of a definite plant species for the same ailment. The FL values indicate the importance of certain plant species for particular purpose. All of the reported ailments grouped into major classes for the calculation of FL values [19]. FL value was estimated by using the formula FL = Ip/Iu × 100, where Ip represents the number of respondents who reported the medicinal plants utilization for a particular ailment and Iu is the total number of respondents who mentioned the same plant for any ailment [22]. It is assumed that those medicinal plants which are used frequently by most respondents for the same disease category are more likely to be biologically active plants [23].

### 2.7. Preference Ranking (PR)

Preference ranking technique was carried out selecting fifteen key respondents following standard method [24] to identify the most preferred species used for treating the most commonly reported gastrointestinal disease in the area.

### 2.8. Direct Matrix Ranking (DMR)

Data on use diversity of multipurpose medicinal plants was gathered using DMR practice [24]. Total 15 key informants were selected on the basis of their strong traditional knowledge regarding medicinal plants. Informants selected for DMR were asked to give use values (5 = best, 4 = very good, 3 = good, 2 = less used, 1 = least used, and 0 = not used) to each species. The values (average scores) given to each medicinal plant were summed up and ranked.

### 2.9. Collection and Preservation of Reported Medicinal Plants

For the collection of reported medicinal plants of the study area, field trips were made with local informants. For further processing, these collected medicinals were brought to the laboratory of Kohat University of Science and Technology (KUST), Kohat, Pakistan, and processed using normal method [25]. The scientific names, family names, and names of publication authors were corrected according to the flora of Pakistan and software index kewensis [26, 27]. Plants were dried and pressed on herbarium sheets and deposited at the Herbarium of Department of Botany KUST, Kohat, Pakistan.

## 3. Results

**Figure 2:**
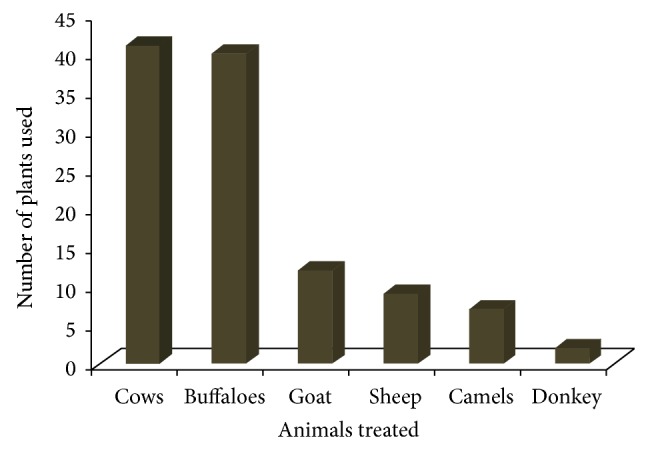
Number of plants used to treat different domestic animals.

The present study revealed ethnoveterinary medicine of 43 plants that belong to 26 families for the treatment of different types of livestock ailments (Table 1). Traditional healers mostly used herbs (56%) for herbal preparation followed by trees (30%) and shrubs (19%) (Table 2). Almost all plant parts were being used for medicinal purposes but seeds (29%) and leaves (22%) were found to be the most frequently used plant part followed by whole plant (12%), fruits (10%), and stem (8%) (Table 2). Inhabitants of the region used these plants for the treatment of different types of domestic animals like buffaloes, cows, goat, sheep, camels, and donkey. A total of 41 plants were found to be used against treatment of cows ailments followed by 40 plants against buffalo's ailments, 12 for goats, 9 for sheep, 7 for camels, and 2 for donkeys (Figure 2).

Gastrointestinal infections were found to be the most common infections in domestic animals and a total of 23 plants were used against them followed by 20 plants that are used against mastitis (Table 3). Herbal preparations were mostly formulated in the form of decoction, powder, and crushing. Different types of vehicles were found to be used for preparation and administration of plant recipes like sugar, flour, water, and milk (Table 1). The most common route of administration was oral (87%) followed by dermal (11%) and only single species is administered through nasal pathway (Table 2). Recovery time of majority of the recipes was three to seven days.

Informant consensus results have shown a high degree of consensus for gastrointestinal (0.81), mastitis (0.82), and dermatological infections (0.80) (Table 3). Eye diseases and reproductive problems scored Fic score (1.00) because only one plant is used against them. The highest plant use citation was for gastrointestinal (122) followed by mastitis (108). The present study revealed 5 medicinal plants having high FL value (Table 4).* Curcuma longa* ranked first score highest FL value (66%) followed by* Trachyspermum ammi* that ranked second (58%),* Foeniculum vulgare* that ranked third (57%), and* Azadirachta indica* that ranked fourth (54%).

Preference ranking (PR) exercise with selected 15 key respondents (randomly selected) for those medicinal plants that were reported to be used to treat diarrhea, the most commonly reported gastrointestinal disorder, showed that* Zingiber officinale, Punica granatum, Triticum aestivum, Gossypium hirsutum*, and* Withania coagulans* were most preferred species for the treatment of diarrhea (Table 5).

DMR implemented on six medicinal plants revealed which medicinal plants are more under pressure in the investigated region. According to the results* Morus alba* ranked first,* Melia azedarach* ranked second, and* Tamarix aphylla* ranked third (Table 6). The present results also showed that these medicinal plants were more exploited for medicinal, fodder, fuelwood, and agricultural purposes (Table 6).

Informants ethnographic data revealed that majority of the informants were male (78%) followed by female (22%). Among 115 informants majority of the informants (36.5%) were aged between 51 and 60 years. A large proportion of respondents (65.2%) were illiterate followed by 13% that were having only secondary education. Females of the region interviewed were housewives while majority of the male (43.7%) were farmers followed by labor (13.9%) (Table 7).

## 4. Discussion

### 4.1. Traditional Use and Medicinal Plants Diversity

The native people of D. I. Khan region rely on livestock as a major support to their livelihoods, employment, crop production, and transport and for generating revenue to sustain life. Documentation of indigenous knowledge on occurrence, prevention, and control of different livestock diseases and medicinal plants used for their treatment is very important for designing and implementing health improvement strategies. The major factor behind using medicinal plants against livestock infections might be related to the availability of few veterinary clinics and veterinarians mostly in the city area of the region that is insufficient for the treatment of such a high abundance of livestock population. Majority of the people are living in villages which are not easily accessible to the rare modern veterinary services which are also known for their high prices absolutely unaffordable to the people living there due to their poor economic status. The present study revealed that the region consists of 43 medicinal plants belonging to 26 families being traditionally used for the treatment of different livestock ailments. These results provide an indication that study area has rich diversity of ethnoveterinary medicinal plants and indigenous knowledge associated with conventionally used species. Similar results have also been found in other areas of Pakistan and other countries [2, 5, 28–30]. This comparison confirms the richness of the area in diversity of ethnoveterinary plants.

### 4.2. Preferred Ethnoveterinary Plant Families in the Region

Local people of the region use a total of 26 plant families traditionally against livestock infections. Families with the highest number of ethnoveterinary plants are Poaceae, Solanaceae, and Liliaceae. Family Poaceae does not usually position highly in terms of species richness in ethnoveterinary studies [5, 31, 32]. In spite of this reality the family is the most diversified family of the region and the fourth universal diversified family [33]. The present study is in line with the finding presented by Benítez et al. [34] in Spain. Solanaceae and Liliaceae families are the most important families in ethnoveterinary studies [1, 35], so their high ranking in the present study is not surprising. Moreover, the wide utilization of species from these families might relate to strong traditional beliefs and the presence of effective bioactive ingredients against livestock ailments [36].

### 4.3. Life Form of Plants

Inhabitants of the region mostly prefer herbs and trees in herbal preparations for the treatment of their animals. Majority of the investigators have also found herbaceous plants dominancy for ethnoveterinary and ethnomedicinal purposes in Pakistan and elsewhere [37–39]. The recurrent use of tree species in the region might be endorsed to their easy availability in the immediate environment with great abundance. The high abundance of tree species in the region also indicates the fact that there might be tree-rich forests areas in the past. This fact might be the factor responsible for the indigenous knowledge of the people and invented as a result of recurrent trial and errors. The higher utilization of tree species for ethnoveterinary purposes is in line with the finding recorded in other studies [40, 41]. The present findings are in contrast with other studies conducted elsewhere in which use of high number of shrubs is recorded [42, 43]. However, the discrepancy in domination of growth forms of medicinal plants used among variety of groups in the country could be associated with different geography and ecological diversity and unique indigenous knowledge of different communities. It was also found that majority of the medicinal plant harvesting is undertaken from noncultivated sources while some of the medicinal plants, for example,* Allium sativum* and* Allium cepa,* are being domestically cultivated in the region. Cultivation of these species is not for the purpose of medicinal use but primarily for food and spice. Native healers lack interest to domesticate wild medicinal plants used to treat specific illness in the region. Overexploitation and reliance on wild resources and dwindling of wild habitats due to increasing human population is a serious risk to the medicinal plant resources in the area. Similar tendency of medicinal plants overexploitation from the wild was also reported [44, 45].

### 4.4. Plant Parts Used to Treat Livestock Ailments

The present results revealed that seeds and leaves are the most frequently harvested plant part for the preparation of different medicinal recipes of livestock. Although the highest use of leaves is reported from numerous ethnoveterinary studies [39, 46] the highest utilization of seeds is rare. A good reason for using leaves and seeds for the treatment of livestock ailments might be associated with their highest bioactivity due to the presence of different secondary metabolites in leaves and ripened seeds. Major difference between human and veterinary medicines is the lack of care during plant part harvesting. For human treatment traditional healers select and collect plant part very vigilantly, while for ethnoveterinary uses people collect the whole shoot or stem rather than just collecting only leaves. From an ecological point of view, herbal formulation that involves stem, whole plant, bulb, roots, and so forth has effect on plant life or survival of the mother plant [38]. Leaves and seeds are the renewable parts of plant and their collection does not result in the fatality of the mother plants. During leaves harvesting some of the leaves remain on the parent plant that carries on essential physiological processes of plant. Protection from harms due to ethnoveterinary collection may be less important when the collected plant parts are renewable.

### 4.5. Ethnoveterinary Medicines Preparation

Main form of remedy preparation in the study area is decoction. It has already been identified that decoction process produces complete extraction of therapeutic compounds. Similar findings are also reported from other regions [5, 34, 47]. In the studied region mostly the recipes are formulated using single plant species. Polyherbal preparations are not much followed in the region and that might be due to variation in indigenous knowledge of different communities. Monotherapy preparation involving single medicinal plant has also been reported from the other regions of Pakistan [11, 15, 48]. The most common route of administration in the study area is oral followed by dermal. The highest number of plants taken through oral route indicates the presence of large number of internal livestock infection in comparison with external. Yigezu et al. [41] reported that oral and dermal routes are considered the most effective methods due to their immediate physiological reaction with the pathogens and escalating the healing capacity of the medicine. Different vehicles like sugar, milk, water, flour, common salt, and so forth are being used in the study region in formulation of ethnoveterinary recipes. These vehicles help in reducing the bitter taste of the herbal remedy and ensure intake of complete dosage of medication. Dose of the ethnoveterinary recipes was documented for some of the plants while for majority of the plants there was no standardized dose and that might be due to the fact that the dose might be increased or decreased depending upon the disease severity, size, and body condition of the animal. Lack of standard dose of ethnoveterinary preparation is one of the major shortcomings of traditional health care system in Pakistan. Recovery time for most of the ailments was reported in range of 3–7 days but according to traditional healer statements recovery of the animals is usually judged when animals restart their proper feeding and daily activities normally. Similar findings are also reported by other ethnoveterinary studies conducted elsewhere [5, 49].

### 4.6. Animals Treated in the Region

Local people of the region are greatly dependent on livestock for variety of purposes such as food, income, transport, and crop production. In the study area people rear different types of animals including cows, buffaloes, goats, sheep, donkeys, and camels. Majority of the animals are involved in milk, meat, and crop production while an animal like donkey is mostly used for transport purposes. The domestication of variety of animals in the investigation might be associated with their socioeconomic status. Domestication of these animals is very crucial for the inhabitants of the region as it is associated with their monthly income. Similar results have also been conducted by van der Merwe et al. [50] and Benítez et al. [34].

### 4.7. Livestock Ailments Treated in the Region

Traditional healers of the region have sound knowledge to maintain their livestock health by using different ethnoveterinary medicines. Gastrointestinal and mastitis are the most common animal diseases reported in the area. It has already been identified that gastrointestinal infections and mastitis are very common in lactating animals due to the consumption of poor quality of fodder couple with different environmental factors [51]. Informant consensus results also revealed highest citation report for gastrointestinal and mastitis. These results give an indication about the bioactivity of medicinal plants used to treat these ailments. According to Heinrich et al. [20], high Fic values are very useful in the selection of specific plants for further search of bioactive compounds. Eye infections and reproductive disorders scored highest Fic value (1.00 each) because the number of taxa (Nt) used to treat these infection is only one. This indicates that species should be subjected to further* in vitro* screening that could lead toward the extraction of some novel compounds against these problems. Extensively used medicinal plants for specific ailments always score highest fidelity level. Present study determined different plants like* Curcuma longa*,* Trachyspermum ammi*,* Foeniculum vulgare, Brassica campestris, and Azadirachta indica* scored high FL value and could be further searched for their* in vitro* investigation and efficacy.

### 4.8. Gastrointestinal Ailments

Majority of the plants in the region are used to treat different types of gastrointestinal problems of the livestock like diarrhea, expulsion of worms, constipation, abdominal pain, and so forth. Stomach diseases are common and may be due to independent foddering of animals in meadows where they eat different types of herbs, sometimes poisonous weeds as well. Mostly symptoms of gastrointestinal ailments are related to those of humans. Mostly herbs are used in treatments and given orally to cattle, for example, crushed stem of* Allium sativum* boiled in water and made decoction to treat gastrointestinal problems of goats and sheep. Phytochemical screening of* Allium sativum* confirmed the presence of Allicin that is responsible for its action against gastrointestinal pathogens [52]. Saravanan et al. [53] also reported* in vitro* validation of different extracts of* Allium sativum* against common gastrointestinal bacteria* Escherichia coli* and* Salmonella typhi*. Strained water of* Cassia fistula* fruit is used in stomach problems of cows and buffaloes. Bhalodia et al. [54] reported that hydroethanol and chloroform extract of* Cassia fistula* fruit exhibit strong anti-*Escherichia coli* activity that might be due to the presence of different classes of compounds, that is, terpenoids, saponins, steroids, phenolic compounds, anthraquinone, and glycosides identified in the tested extracts. Some plants are used in mixture with other plants or additives for herbal formulations, for example, seeds of* Foeniculum vulgare* are mixed with different additives and root of* Glycyrrhiza glabra* is mixed with flour, and used for stomach problems of cows, buffaloes, and camels. Manonmani et al. [55] studied* Foeniculum vulgare* seeds* in vitro* and found that aqueous extract of seeds exhibits strong inhibitory activity against gastrointestinal pathogens such as* Escherichia coli*,* Salmonella typhi,* and* Bacillus cereus*. Ether, acetone, and chloroform extract of* Glycyrrhiza glabra* showed significant antibacterial activity that might be due to the presence of biological active compound glycyrrhizin [56]. Leaves of* Morus alba* are crushed to make powder and mixed in milk to form suspension given orally for 4 days in case of constipation of cows and buffaloes. Devi et al. [57] confirmed the antibacterial activity of leaves of* Morus alba* that might be due the presence of different phytocompounds isolated from the leaves such as rutin, quercetin, and apigenin [58]. Fruit cover of* Punica granatum* is cut into thin pieces and mixed with wheat flour given orally to treat diarrhea. Different classes of phytochemicals such as phenolic compounds and flavonoids have been identified from the peel of* Punica granatum* that might be responsible for its activity against gastrointestinal pathogens [59]. The pharmacological justification of these plants ensures strong validity and reliability of traditional knowledge of the respondents.

### 4.9. Mastitis

In the study area mastitis is the second major disease of domestic animals. Mastitis is common in other areas of Pakistan as well as throughout the world [6]. Mastitis is an infectious disease of udder which is mostly caused by* Staphylococcus aureus* and* Streptococcus agalactiae* due to which milk production rate is decreased. Other bacteria encountered include* Corynebacterium pyogenes*,* Klebsiella* spp.,* Mycobacterium* spp., and* Brucella* spp. Many* in vitro* activities by different plants have been proved for mastitis and give pharmacological proof of traditional validity of plants; for example, increasing concentration of* Zingiber officinale* inhibits the growth of* S. aureus* and* Streptococcus agalactiae* [60]. In the study area* Zingiber officinale* is also used to treat mastitis and is very effective therapy for cows and buffaloes. Traditionally, 150 g of* Allium cepa* alone or combined with* Trachyspermum ammi* is given orally for five days to treat mastitis.* Allium sativum* is also a preferred plant in other regions of Pakistan against mastitis [61].* In vitro* it is proved that* Allium* vegetables have broad antibiotic spectrum against both gram positive and gram negative bacteria.* Allium sativum* is effective to those strains also which show resistance to antibiotics [62].

### 4.10. Dermatological Problems

Scabies and allergy are commonly reported in the area and 4 plants are used in treatment of dermatological ailments. Pieces of* Aloe barbadensis* leaves are mixed with common salt and given orally to all cattle. Aqueous extract of leaves of* Aloe barbadensis* has already been proved* in vivo* for their significant activity against skin inflammation in albino Wistar rats [63]. Leaves of* Tamarix aphylla* are crushed and made into poultice and are applied on skin wounds. This plant is also being used for dermatological infection in India to cure buffaloes [64].* Citrullus colocynthis* is most effective in all kinds of dermatological problems and is given orally as well and is made into poultices. Seed and fruit extracts of* Citrullus colocynthis* showed strong anti-inflammatory activity in rats without any side effects [65]. Leaves of* Azadirachta indica* have also application in skin abscesses.

### 4.11. Respiratory Infections

Mostly respiratory disease symptoms are similar to those of humans and are so easily diagnosed. Cough is common in cattle in the study area and two plants are used for cough. Crushed stem of* Glycyrrhiza glabra* is mixed with oil, wheat flour, and gurr or sugar to make halwa and 250 g is given orally for 3 days. Kushwah et al. [66] studied the ethanolic extract of stem of* Glycyrrhiza glabra* and confirmed its antimicrobial activity. The phytochemical screening of ethanolic extract revealed the presence of alkaloids, saponins, carbohydrates, tannins, and steroids. Seeds of* Hordeum vulgare* are roasted and grinded to make powder. Powder is mixed with water and given to animals for 7 days. This plant has already been identified as potential antimicrobial agent due to the presence of alkaloids, saponins, volatile oil, saponins, and terpenes [67].

### 4.12. Reproductive and Eye Diseases

Conjunctivitis is simply an inflammation of the soft tissues surrounding the eye and eyelids. It is common in study area in the months of April-May. Ruminants such as cattle and goats affected with conjunctivitis will have reddening of the eyeball and swelling of the inner lining of the eyelid. These animals will have an increased sensitivity to sunlight which is demonstrated by “squinting” or closing their eyes in bright sunlight. There will usually be a discharge from the eye as well. In the area only one plant* Albizia lebbeck* is reported for eye disease and is very effective. Only one plant* Brassica campestris* is used for placenta retention during delivery. It is observed that there is lack of traditional uses of plants in case of reproductive disorders because mostly death of cows and buffaloes happened during delivery in the study area but at this stage respondents mostly prefer veterinary technicians.

### 4.13. Multipurpose Ethnoveterinary Plants

DMR results showed that six species out of 43 have variety of other nonmedicinal uses. The present results showed that* Morus alba*,* Tamarix aphylla*,* Melia azedarach*,* Cassia fistula*,* Withania coagulans,* and* Azadirachta indica* are the multipurpose species of the region. These species are mostly overharvested for medicinal, fodder, fuel, agriculture, and construction purposes. The high utilization of these species for fodder and fuel purposes might relate with the high dependency of the people on livestock and unavailability of modern fuel resources in the region, respectively. Our findings are also in line with the study by Barkatullah et al. [68] carried out in Malakand district. They found that in the absence of gas supply and other fuel types in the area, the local people extensively use tree species as fuelwood. Income status of the locals is low due to the fact that low literacy ratio in the study area might be another reason of their higher dependency on medicinal plants for different income generating or compensating purposes. Therefore, it is necessary to take serious concern regarding the conservation of such valuable ethnoveterinary medicinal plants of the region.

## 5. Conclusions

The present study concluded that study region has a great reservoir of ethnoveterinary medicinal plants and locals of the region have tremendous traditional knowledge to utilize these plants for the treatment of their livestock. Plants scoring high Fic and FL value should be further tested for their phytochemical and pharmacological investigation. Protection measures should be adopted for the conservation of multipurpose and other medicinal plant species. Young generation should be mobilized toward learning ethnoveterinary practices before its extinction.

## Figures and Tables

**Table 1:** Ethnoveterinary uses of medicinal plants of Dera Ismail Khan.

Plant name/families/voucher number	Local name	Habit	Life-span	Part used	Disease type	Animal treated	Recipe	Dosage (g)	Route of administration	Recovery (days)
*Albizia lebbeck* L., Mimosaceae, KUH-721	Sirin	T	P	L	Eye diseases	Cows, buffaloes, sheep, goat, donkey, camels	Leaves are boiled in water and used for eye diseases in animals	3-4 drops	Dermal	3-4

*Allium cepa* L., Liliaceae, KUH-722	Piaz	H	A	Bu	Mastitis	Cows, buffaloes	1000 g of onion alone or combination of onion and ajwain is used	150	Oral	5

*Allium sativum *L., Liliaceae, KUH-723	Lehsan/thoom	H	A	St	Bloody diarrhea	Sheep, goat	Decoction	1-2 glass	Oral	10
				Bu	Mastitis	Cows, buffaloes	Plant part grinded and mixed with butter	150	Oral	7

*Aloe barbadensis* Mill., Liliaceae, KUH-724	Kuwar gandal	H	P	L	Scabies, stomach troubles	Cows, buffaloes, sheep, goat, donkey, camels	Pieces of spineless leaves are mixed with common salt	2 leaves	Oral	10

*Amomum subulatum *Roxb., Zingiberaceae, KUH-725	Baree Ilaichee	T	P	F	Mastitis	Cows, buffaloes	Grinded and mixed with ajwain, sarsoon oil, and salt	25	Oral	3

*Azadirachta indica* L., Meliaceae, KUH-726	Neem	T	P	L	Scabies, skin abscesses	Cows, buffaloes, goats	Fresh leaves are crushed and mixed with water. The water is strained and then given to animals	Daily one glass	Oral	7

*Brassica campestris *L., Brassicaceae, KUH-727	Sarsoon	H	A	So	Mastitis	Cows and buffaloes	500 mL of seeds oil is given daily	500 mL	Oral	10
				S	Placenta retention	Cows, buffaloes	Decoction is made by mixing lassi, crushed seeds, and some amount of soap	As needed	Oral	1
				S	Placenta retention	Cows, buffaloes	Mixture of sugar, crushed seeds, and milk is given	As needed	Oral	1
				S	Mastitis	Cows/buffaloes	Seed oil	150	Oral	4-5
				S	Placenta retention	Cows, buffaloes	Seeds are crushed and given in powder form	150	Oral	3

*Capsicum annuum *L., Solanaceae, KUH-728	Lal mirch	H	A	Wh	Mastitis	Cows, buffaloes	Plant is crushed and mixed with wheat	As needed	Oral	8

*Cassia fistula *Mill., Caesalpinaceae, KUH-729	Amaltas	T	P	F	Mucal diarrhea, muscles and joints pain	Cows, buffaloes	Fruit is broken and boiled in water. The water is strained and then used	As needed	Oral	3–5

*Centratherum anthelmisticum *L., Asteraceae, KUH-730	Kala Zeera	H	A	S	Mastitis, stomach troubles	Cows and buffaloes	50 g of seeds is mixed in wheat flour and common salt	50	Oral	5

*Citrullus colocynthis *L., Cucurbitaceae, KUH-731	Karthuma	H	A	R	Skin diseases	Buffalo, cows, goat	Poultice	As needed	Dermal	3
				F	Mastitis	Cows and buffaloes	Grind fruit and mixed with khal or flour	100	Oral	3

*Citrus limon* L. Osbeck, *Rutaceae,* KUH-732	Nimbu, Lemon/Lemo	S	P	F	Mastitis, stomach troubles	Cows, buffaloes, sheep, goat	Fruit juice is mixed with sugar to make paste		Oral, dermal	15

*Citrus reticulata* Blanco, Rutaceae, KUH-733	Malta, sangtara	T	P	L	Diarrhea	Cows, buffaloes, sheep, goat, camels	Dry leaves are boiled in water to make juice	As needed	Oral	2
				L	Mastitis	Cows, buffaloes, goat	Poultice	As needed	Dermal	5

*Cleome brachycarpa* Vahl., Capparaceae, KUH-734	Gandi booti	H	A/P	Wh	Malash	Cows, buffaloes, goats	Crushed and mixed with wheat flour	As needed	Oral	4

*Cuminum cyminum *L., Asteraceae, KUH-735	Sufaid zeera	H	A	S	Mastitis	Cows and buffaloes	1 kg of seeds is crushed	1000	Oral	6

*Curcuma longa *L., Zingiberaceae, KUH-736	Haldi	H	P	R	Mastitis	Cows, buffaloes, sheep, goat	Roots and sugar are grinded to make powder	30	Oral	7

*Echinops echinatus *Roxb., Asteraceae, KUH-737	Mastara	S	A	St	Garmi/malash	Cows/buffaloes	Crushed and mixed with flour and made into balls	50	Oral	10

*Eruca sativa *Mill., Brassicaceae, KUH-738	Ussoon	H	A	S	Malash	Cows and buffaloes	Seeds crushed and made into small balls and mixed with oil	70	Oral	3

*Fagonia cretica* L., Zygophyllaceae, KUH-739	Dhamaan	S	B	Wh	Liver tonic	Cows, buffaloes,	Decoction	2-3 glass	Oral	4-5

*Foeniculum vulgare *Mill., Apiaceae, KUH-740	Saunf	H	P	S	Indigestion	Cows/buffaloes/camels	Powder is made and called *Phaki* by mixing the plant part with meetha soda, malathi, and ajwain	100	Oral	After one day till 10 days
	Saunf			S	Stomach troubles	Cows and buffaloes	Crushed seeds are mixed with wheat flour, black salt, sodium bicarbonate, and common salt	As needed	Oral	2
	Saunf			S	Mastitis	Cows and buffaloes	50 g of seeds is roasted and mixed in 125 mL of vegetable oil	As needed	Oral	4

*Glycyrrhiza glabra* L., Fabaceae, KUH-741	Malathi	S	P	St	Cough	Cows, buffaloes, goat, sheep	Crushed stem is mixed with oil, wheat flour, and gurr or sugar to make halwa	250	Oral	3

*Gossypium hirsutum* L., Malvaceae, KUH-742	Cotton buds	S	A	B	Bloody diarrhea	Cows, buffaloes	Buds are crushed and mixed with sarson oil and made into small balls of 100 g	100	Oral	4-5

*Hordeum vulgare,* Poaceae, KUH-743	Barley, Jow	H	A	S	Cough	Cows, buffaloes	Seeds are roasted and grinded to make powder. Powder is mixed with water and given to animals	One bucket	Oral	7

*Ipomoea carnea *Jacq., Convolvulaceae, KUH-744	Wirnra	S	P	L	Malash/fever	Cows, buffaloes, sheep, goat, camels	Dried leaves are crushed and made into safoof		Oral	4-5

*Mangifera indica *L., Anacardiaceae, KUH-745	Mango/Aamb/Aam	T	P	S	Mucal diarrhea, muscles pain	Cows, buffaloes	Crushed seeds are given	As needed	Oral	1-2

*Melia azadirachta *L., Meliaceae, KUH-746	Dhrek	T	P	L	Stomach troubles	Cows, buffaloes	Leaves are crushed with sugar to make powder	25	Oral	5

*Morus alba *L., Moraceae, KUH-747	Toot	T	P	L	Constipation	Cows, buffaloes	Leaves are crushed to make powder and mixed in milk to form suspension	As needed	Oral	3-4

*Nigella sativa *L., Ranunculaceae, KUH-748	Kaoolnji	H	A	S	Mastitis	Cows and buffaloes	Seeds are boiled in water to make suspension	As needed	Oral	4

*Oryza sativa *L., Poaceae, KUH-749	Chawal	H	A	S	Mastitis	Cows and buffaloes	Seeds are boiled in milk to make suspension		Oral	2

*Peganum harmala *L., Zygophyllaceae, KUH-750	Harmal	H	A	Wh	Mastitis	Cows and buffaloes	Fumigation of harmal seeds under the affected udder at evening time; fresh leaves are mixed with chara and given	As needed	Nasal, oral	5

*Piper nigrum *L., Piperaceae, KUH-751	Kali mirch	T	P	S	Mastitis	Cows, buffaloes	Mixture of kali mirch and wheat flour is given	As needed	Oral	7

*Portulaca oleracea* L., Portulacaceae, KUH-752	Loonrak	H	A	Wh	Mastitis	Cows, sheep	Full plant is given	As needed	Oral	

*Punica granatum* L., Lythraceae, KUH-753	Anar	T	P	F	Bloody diarrhea	Buffaloes, cows, goat	Fruit cover is cut into thin pieces and mixed with wheat flour	250	Oral	2

*Rosa indica *L., Rosaceae, KUH-754	Saunf, gulab, podina, ajwain	H	P	E	Constipation, abdominal swelling	Cows, buffaloes, goat, sheep	Extract of saunf, gulab, podina, and ajwain is mixed and given after half an hour	Equal amount of all these extracts	Oral	2
	Rose, Gulab			P	Mastitis	Cows and buffaloes	Petals are boiled in milk	As needed	Oral	7

*Saccharum officinarum *L., Poaceae, KUH-755	Kamad/Ganna	S	A	St	Mastitis	Cows and buffaloes	Stem is crushed to obtain juice extract	As needed	Oral	7

*Sesamum indicum *L., Pedaliaceae, KUH-756	Til	H	A	So	Mastitis, Malash	Cows, buffaloes	Mixture of oil and milk or lassi	As needed	Oral	7

*Tamarix aphylla *L., Tamaricaceae, KUH-757	Khagal	T	P	L	Killing of worms in skin wounds	Cows, buffaloes	Leaves are crushed to make poultice	As needed	Dermal	5

*Thymus serpyllum *L., Lamiaceae, KUH-758	Jangli Podina	H	B	L	Mastitis	Cows, buffaloes	Poultice		Dermal	7

*Trachyspermum ammi *L., Apiaceae, KUH-759	Ajwain	H	A	S	Stomach troubles, mastitis, abdominal swelling, allergy	Cows, buffaloes, goat, camels	Crushed seeds are mixed with wheat flour and common salt	80–100	Oral	4

*Triticum aestivum *L., Poaceae, KUH-760	Gandam/kanak	H	A	S	Diarrhea	Cows and buffaloes	Seeds are crushed to make fine powder and make small balls shape	100	Oral	3

*Withania coagulans*L., Solanaceae, KUH-761	Akri/paneer	S	P	L/F	Diarrhea	Cows, buffaloes, sheep, goat	Fruit is crushed and mixed with oil and salt	As needed	Oral	3-4

*Xanthium strumarium* Linn., Solanaceae, KUH-762	Kandari	H	A	Wh	Malash	Camels	Powder	As needed	Oral	10

*Zingiber officinale *Roscoe., Zingiberaceae, KUH-763	Sund/Adrak	H	P	Rh	Mastitis, bloody diarrhea	Cows and buffaloes	Rhizome and sugar are grinded to make fine powder and give with sugar	130	Oral	5

Habit: H: herb; T: tree; S: shrub. Life span: A: annual; B: biennial; P: perennial. Plant part: L: leaf; Rh: rhizome, Wh: whole plant, F: fruit; S: stem; So: seed oil; E: extract; Bu: bulb; Fl: flower.

**Table 2:** Habit, life-span, and parts used of ethnoveterinary plants.

General attributes	Total plants	%age
Habit		
Herb	24	56
Tree	11	30
Shrub	8	19
Life-span		
Annual	21	49
Perennial	20	47
Biennial	2	4
Part used		
Seed	15	29
Leaves	11	22
Whole plant	6	12
Fruit	5	10
Stem	4	8
Bulb, rhizome, root, seed oil	2	4
Buds, extract	1	2
Route of administration		
Oral	48	87
Dermal	6	11
Nasal	1	2

**Table 3:** Informant consensus factor.

Disease categories	Number of taxa (Nt)	Number of used reports (Nur)	*F*_IC_
Eye disease	1	3	1
Reproductive	1	10	1
Respiratory	2	4	0.66
Dermatological	4	16	0.80
Mastitis	20	108	0.82
Gastrointestinal	23	122	0.81
Muscular	2	6	0.8

**Table 4:** Fidelity level (FL).

Number	Plant species	Disease category	Ip	Iu	FL%
1	*Curcuma longa *	Mastitis	10	15	66.6
2	*Trachyspermum ammi *	Gastrointestinal	17	29	58.6
3	*Foeniculum vulgare *	Gastrointestinal	11	19	57.8
4	*Azadirachta indica *	Dermatological	6	11	54.5
5	*Brassica campestris *	Reproductive	10	20	50

**Table 5:** Preference ranking of ethnoveterinary medicinal plants used against treatment of diarrhea.

Antidiarrheal medicinal plants	Informants labeled A to O															Total score	Rank
	A	B	C	D	E	F	G	H	I	J	K	L	M	N	O		
*Allium sativum *	4	6	4	5	4	3	6	5	4	5	3	4	4	4	4	65	6
*Cassia fistula *	3	2	1	3	4	3	5	2	2	5	5	3	2	3	2	45	8
*Citrus reticulate *	4	5	3	5	6	2	4	2	5	3	5	3	2	4	3	56	7
*Gossypium hirsutum *	5	4	4	6	4	4	5	6	5	6	5	6	4	3	5	72	4
*Mangifera indica *	3	5	4	7	5	2	4	3	4	6	4	6	4	5	3	65	6
*Punica granatum *	6	7	5	6	7	4	4	5	5	4	6	7	4	7	4	81	2
*Triticum aestivum *	4	4	4	4	3	5	6	4	6	7	6	6	7	5	4	75	3
*Withania coagulans *	2	3	2	6	6	6	6	4	5	4	5	5	6	5	5	70	5
*Zingiber officinale *	7	7	7	7	6	7	4	7	7	5	7	7	6	5	7	96	1

Table scores indicate ranks given to medicinal plants based on their efficacy. Highest number (7) for the medicinal plant which informants thought was most effective in treating diarrhea and the lowest number (1) for the least-effective plant.

**Table 6:** Direct matrix ranking.

Use diversity	*C. fistula *	*M. azedarach *	*T. aphylla *	*W. coagulans *	*M. alba *	*A. indica *	Total	Rank
Fodder	4	4	4	4	4	4	24	3
Fuel	5	5	5	3	5	4	27	2
Construction	2	3	3	1	4	3	16	4
Agriculture	3	3	2	1	4	3	16	4
Medicinal	4	5	5	5	5	5	29	1

Total	18	20	19	14	22	19		
Rank	4	2	3	5	1	3		

Based on used criteria (5 = best; 4 = very good; 3 = good; 2 = less used; 1 = least used; 0 = no value).

**Table 7:** General information about respondent interviewed.

	Total	Percentage
Gender		
Male	90	78
Female	25	22
Age groups		
30–40	18	15.6
41–50	30	26
51–60	42	36.5
61–70	25	18.2
Educational attainment		
Illiterate	75	65.2
Primary	7	6
Middle	10	8.6
Secondary	15	13
University	8	6.9
Occupation		
Females		
Housewives	25	12.1
Male		
Shopkeepers	14	12.1
Farmers	50	43.7
Labors	16	13.9
Primary teachers	10	8.6
